# Loss of neuronatin promotes “browning” of primary mouse adipocytes while reducing Glut1-mediated glucose disposal

**DOI:** 10.1152/ajpendo.00463.2012

**Published:** 2013-03-12

**Authors:** Valentina Gburcik, Mark E. Cleasby, James A. Timmons

**Affiliations:** ^1^Department of Comparative Biomedical Sciences, Royal Veterinary College, University of London, London, United Kingdom; and; ^2^School of Sports, Exercise and Health Sciences, Loughborough University, Loughborough, United Kingdom

## Abstract

Failure of white adipose tissue to appropriately store excess metabolic substrate seems to underpin obesity-associated type 2 diabetes. Encouraging “browning” of white adipose has been suggested as a therapeutic strategy to help dispose of excess stored lipid and ameliorate the resulting insulin resistance. Genetic variation at the DNA locus encoding the novel proteolipid neuronatin has been associated with obesity, and we recently observed that neuronatin expression is reduced in subcutaneous adipose tissue from obese humans. Thus, to explore the function of neuronatin further, we used RNAi to silence its expression in murine primary adipocyte cultures and examined the effects on adipocyte phenotype. We found that primary adipocytes express only the longer isoform of neuronatin. Loss of neuronatin led to increased mitochondrial biogenesis, indicated by greater intensity of MitoTracker Green staining. This was accompanied by increased expression of UCP1 and the key genes in mitochondrial oxidative phosphorylation, PGC-1α, Cox8b, and Cox4 in primary subcutaneous white adipocytes, indicative of a “browning” effect. In addition, phosphorylation of AMPK and ACC was increased, suggestive of increased fatty acid utilization. Similar, but less pronounced, effects of neuronatin silencing were also noted in primary brown adipocytes. In contrast, loss of neuronatin caused a reduction in both basal and insulin-stimulated glucose uptake and glycogen synthesis, likely mediated by a reduction in Glut1 protein upon silencing of neuronatin. In contrast, loss of neuronatin had no effect on insulin signaling. In conclusion, neuronatin appears to be a novel regulator of browning and metabolic substrate disposal in white adipocytes.

sustained adipose tissue remodeling appears to be instrumental in preventing the development of insulin resistance (IR) and type 2 diabetes in obese subjects (35). It has been proposed that the failure of subcutaneous adipocytes to appropriately store excess calories in obesity causes IR through the resulting inappropriate deposition of triacylglycerol (TAG), diacylglycerols (DAG), and/or ceramides in muscle and liver (reviewed in Refs. 6, 10, 11, 28, 34). However, there are two distinct types of adipose tissue. White adipose tissue (WAT) stores TAG derived from circulating fatty acids and glucose in the form of large lipid droplets and to a lesser extent as glycogen. In contrast, brown adipose tissue (BAT) is characterized by the presence of many smaller lipid droplets and large numbers of mitochondria, in which substrate oxidation is uncoupled from phosphorylation due to the expression of uncoupling protein-1 (UCP1) in the inner membrane of the mitochondria, resulting in the dissipation of energy as heat. In addition, we recently described a novel subtype of white adipocyte, the “brite” (brown-in-white) adipocyte, which upon stimulation by sympathetic or PPARγ agonists, can differentiate into cells which also express UCP1 (8, 25, 26).

Neuronatin is a novel proteolipid that is derived from an imprinted gene situated on the paternal allele (15); genetic variation at the neuronatin locus has been associated with obesity and variation in fat mass in humans (39). In adult rats and humans, neuronatin mRNA is highly expressed in hypothalamic nuclei, where it has a short half-life, and its expression is regulated by feeding/fasting and leptin. Neuronatin is also expressed in pancreatic β-cells, where it modulates insulin secretion in response to glucose. Thus, neuronatin seems to play a role in nutrient sensing in mammals (38, 39). Neuronatin mRNA can be spliced into two variants, generating proteins of 81 (α) and 54 (β) amino acids in length (4, 13). Both variants are predicted to possess a single transmembrane domain and seem to reside in the endoplasmic reticulum (ER) in neurons (12, 18, 24). Neuronatin-α is the isoform preferentially reduced in the β-cells of diabetic rodents (1, 12). Conversely, neuronatin-α appears to be increased in the endothelial cells of blood vessels from obese and diabetic mice (22). Recently Sharma et al. (30) identified neuronatin as a substrate for the E3 ubiquitin ligase malin, an activity that inhibits neuronatin-mediated activation of GS.

We recently showed that subcutaneous neuronatin expression declines with increasing obesity in humans (14), suggesting that the role of neuronatin in adipocytes merited more detailed investigation. However, it is not known which isoform of neuronatin is expressed in adipocytes, and there have been no mechanistic studies published that have explored the role of neuronatin in adipocytes. Given the potential role of neuronatin in regulating adipocyte metabolism, we studied the impact of RNAi-mediated loss of neuronatin expression in subcutaneous primary adipocytes on key indicators of adipocyte phenotype and glucose disposal.

## METHODS

### 

#### Gene expression profiling in human subcutaneous adipose tissue.

Neuronatin gene expression was profiled in subcutaneous abdominal adipose tissue samples from 33 human subjects by using Affymetrix U133 Plus 2.0 gene chips, as previously published (14, 37). These data can be found, along with the transcript profiles, at NCBI (GSE27951). The mean (SD) age, body mass index (BMI), and V̇o_2 max_ for these subjects were, respectively, 46 (13) yr, 32 (7) kg/m^2^, and 31 (13) V̇o_2 max_/kg, with the range in BMI being 21–48 kg/m^2^. Mean BMI for the group “BMI<30” (16 subjects) was 25.1 ± 2.9, whereas mean BMI for the group “BMI>30” (17 subjects) was 37.3 ± 4.6. Data were normalized using MAS5, and the probe set signal (arbitrary units) for neuronatin and uncoupling protein-1 (UCP1) were compared between normal and obese (BMI>30) individuals.

#### Animals, cell culture, and siRNA transfection.

Adipocyte progenitors were isolated from the 129/Sv strain of mice (Harlan UK). In this strain, following cold exposure, UCP1 mRNA levels in subcutaneous adipose tissue approach those in interscapular brown fat, demonstrating that they are capable of robust “browning” (16). White adipocyte precursors (iWA) were isolated from inguinal subcutaneous adipose tissue depots and brown adipocyte precursors (BA) from the pooled interscapular and axillary brown adipose depots of 3-wk-old mice and processed as described previously (25). The pellet of precursor cells was suspended in culture medium and then cultured in six-well plates containing DMEM with 10% (vol/vol) newborn calf serum (Invitrogen), 2.4 nM insulin, 25 μg/ml sodium ascorbate, 10 mM HEPES, 4 mM glutamine, 50 U/ml penicillin, and 50 μg/ml streptomycin, supplemented or not (as indicated) with 1 μM rosiglitazone maleate (EnzoLifeSciences) and 1 μM norepinephrine (NE; Sigma-Aldrich) from the first day in culture (26). The cells were differentiated and cultured at 37°C in an atmosphere of 5% CO_2_ in air with 80% humidity. Where indicated, transfection was performed twice, during differentiation, as previously described (5). Briefly, 24 and 90 h after seeding, 0.32% Lipofectamine 2000 (Invitrogen) and 20 nM siRNA pool targeting neuronatin (Dharmacon) was used (Table 1) in 2.5 ml of serum-containing cell culture medium (without antibiotics). Control cells were treated with Lipofectamine 2000 alone. Mature adipocytes were harvested on *day 6* of culture for further analysis. Before harvesting, they were examined using phase contrast microscopy (on a Leica DMIRB inverted microscope).

**Table 1.** Primers used for qRT-PCR analysis and target sequences for siRNAs used for Nnat knockdown

Gene	Forward Primer (5′-3′)	Reverse Primer (5′-3′)
*Nnat*	CACCCACTTTCGGAACCAT	GCAGGGAGTACCTGAACACCT
*aP2*	CGCAGACGACAGGAAGGT	TTCCATCCCACTTCTGCAC
*UCP1*	GGCCTCTACGACTCAGTCCA	TAAGCCGGCTGAGATCTTGT
*PPARγ*	GAAAGACAACGGACAAATCACC	GGGGGTGATATGTTTGAACTTG
*PGC1α*	GAAAGGGCCAAACAGAGAGA	GTAAATCACACGGCGCTCTT
*Cox8b*	CCAGCCAAAACTCCCACTT	GAACCATGAAGCCAACGAC
*siRNA*	*Target sequence*	
*J-040876-09*	GGGAGCAACCCUCGAGAUA	
*J-040876-10*	AUAUUGUGGUAGUCGCUAA	
*J-040876-11*	GCACAAGAUCCUACCAUGA	
*J-040876-12*	CUGGUUUAAGUGUGCAUUA	

#### RNA isolation and quantitative real-time PCR.

Total RNA was isolated using TRIzol (Invitrogen) according to the manufacturer's protocol. RNA was dissolved in 20 μl of RNAse-free water and quantified using a Nanodrop (NanoDrop Technologies). For determination of target mRNA levels, 1 μg of RNA was reverse-transcribed with a High Capacity cDNA kit (Applied Biosystems) in a total volume of 20 μl. cDNA was diluted 1:10, and 2 μl was added per well of 384-well optical plates. Exon-spanning primers (Invitrogen; Table 1) were premixed with SYBR Green JumpStart Taq ReadyMix (Sigma-Aldrich), and aliquots of 10 μl of this master mix were added to the wells. BioRadThermal cycling conditions were for 10 min at 95°C and 40 cycles of 15 s at 95°C, 30 s at 62°C, and 20 s at 72°C on a CFX384 Real Time System (Bio-Rad). 18S mRNA levels were used for normalization of target mRNA expression quantified according to the manufacturer's protocol in triplicate using the ΔC_T_ method (19).

#### Western blot.

Adipocytes were washed twice in ice-cold PBS and then harvested in a modified RIPA buffer (50 mM Tris·HCl, pH 7.4, 1% Triton X-100, 150 mM NaCl, 1 mM EDTA, 1 mM PMSF, 1 mM Na_3_VO_4_, 1 mM NaF) and a protease/phosphatase inhibitor cocktail (Complete-Mini, Roche Diagnostics). Cells were lysed on ice for 15 min and then centrifuged at 14,000 *g* for 15 min. The concentration of proteins in the supernatant was determined using a BCA protein assay kit (Pierce Biotechnology). The immunoblots were visualized with appropriate primary and horseradish peroxidase-conjugated secondary antibodies and enhanced chemiluminescence (ECL kit, GE Healthcare Life Sciences). The primary antibodies used were as follows: neuronatin (Abcam), Cox4 (Santa Cruz Laboratories), IRS1 (Upstate Cell Signaling Solutions), AS160 (Upstate Biotechnology), β-actin (Sigma-Aldrich), GLUT1 (Millipore) and pS71-ACC, ACC, pT172-AMPK, AMPK, pS473-Akt, Akt, pY612-IRS1, pS21/9 glycogen synthase kinase (GSK)3α/β, GSK3α/β, pS641-glycogen synthase (GS), total GS, and pT642-AS160 (Cell Signaling Technology). Western blots were scanned and analyzed densitometrically using ImageJ software.

#### Analysis of mitochondrial content by MitoTracker green staining.

Mitochondria were labeled using the mitochondria-specific dye MitoTracker Green (Molecular Probes) according to manufacturer's protocol. The final dye concentration was 40 nmol/l, and the incubation time was 30 min prior to visualization. Fluorescent microscopy was performed on live cells with a Leica DMIRB inverted microscope. For fluorescence intensity quantification, cells were grown in 96-well plates, and fluorescence intensity was measured using a Mithras LB 940 Multimode Microplate Reader. Average intensity of the background autofluorescence from the wells not containing cells was subtracted from the main fluorescence intensity values.

#### Oil red O staining.

Lipid droplets in the cells were labeled using Oil red O stain (Sigma). Staining stock solution was prepared as follows: 0.7 g of Oil red O was dissolved in 200 ml of isopropanol, stirred overnight, and then filtered through 0.2-μm filters. A working solution was prepared by mixing six parts of Oil red O stock with four parts of distilled H_2_O and then filtering again through 0.2-μm filters. Cells were fixed in 10% formalin for 1 h, washed with 60% isopropanol, and dried. The working solution was added for 10 min and then removed. Cells were washed immediately four times with deionized water. Cells were then examined using phase contrast microscopy on a Leica DMIRB Inverted Microscope.

#### Glucose uptake assay.

Primary adipocyte cultures were seeded into six-well tissue culture plates and transfected with a siRNA pool targeting neuronatin or used as controls. At *day 6* of differentiation, the cells were starved for 2 h in serum-free DMEM and then treated for 15 min with 10 nM insulin or vehicle. Glucose uptake was initiated by the addition of 2-deoxyglucose (Sigma) to a final concentration of 100 μM and 0.5 μCi 1-deoxy-d-2-[1,2-^3^H(N)]glucose (PerkinElmer) per well in PBS buffer, as previously described (2). After a 10-min incubation at 37°C, cells were washed with ice-cold PBS, lysed using RIPA buffer, and counted for radioactivity in Ultima Gold scintillation cocktail (PerkinElmer) using an LS6500 Multi-Purpose Scintillation Counter (Beckman Coulter). Non-carrier-mediated uptake was determined in the presence of 10 μM cytochalasin B (Sigma-Aldrich) and deducted from the totals, which are expressed per unit protein concentration, measured using a BCA protein assay kit.

#### Glycogen synthesis assay.

Primary adipocyte cultures were starved on *day 6* of differentiation for 2 h in serum-free DMEM (containing 4,500 mg/l glucose). The medium was then removed, and 2 ml of DMEM containing 0.5 μCi/ml d-[^14^C(U)]glucose (PerkinElmer) ± 10 nM insulin was then added to each well. The cells were incubated for 2 h at 37°C, washed three times with ice-cold PBS, and lysed with RIPA buffer, and the extracts were heated at 100°C for 10 min. An aliquot was then taken for protein concentration measurement using the BCA method. Glycogen was precipitated using 600 μl of ice-cold ethanol per extract at 4°C overnight, and then at −20°C for 30 min, in the presence of unlabeled glycogen. After centrifugation, pellets were dissolved in 400 μl of water by heating at 60°C for 20 min, and activity was determined using a Scintillation Counter.

#### Statistical analysis.

Statistical analysis for qPCR assays was performed using two-way ANOVA with repeated measures (RM) on the raw expression values (ΔC_T_), reflecting independent experiments, using GraphPad Prism 6, followed by Newman-Keuls multiple comparison tests where indicated. Data from iWA and BA were analysed separately. Statistical significance in glucose uptake assays, glycogen synthesis assays, and Western blotting was determined using RM two-way ANOVA followed by the Bonferroni multiple comparison test. Levels of significance are labeled on the figures as follows: ***P < 0.001, **P < 0.01, *P < 0.05.

## RESULTS

### 

#### Obesity is associated with reduced neuronatin expression in humans.

**Fig. 1.**
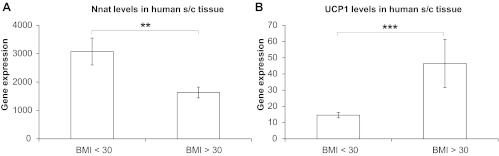
Gene expression profile of neuronatin (Nnat) and UCP1 in human subcutaneous tissue. Neuronatin (*A*) and UCP1 (*B*) expression levels from 33 samples of human subcutaneous tissue, obtained using the Affymetrix U133 Plus 2.0 platform, are shown according to BMI. Mean BMI for the group “BMI<30” (16 subjects) was 25.1 ± 2.9, whereas mean BMI for the group “BMI>30” (17 subjects) was 37.3 ± 4.6. UCP1 data were replotted from Ref. 37. Statistical analysis was performed on log-transformed expression values for neuronatin and UCP1 using repeated-measures (RM) 2-way ANOVA followed by Bonferroni post hoc test.

Neuronatin expression in subcutaneous white adipose tissue was reduced in the obese subjects (BMI>30) (Fig. 1*A*), based on categorical analysis of the previously published raw gene chip data. We previously reported a positive correlation between UCP1 expression and BMI from the same microarray analysis (37) and present it in a different format in Fig. 1*B*.

#### Primary adipocytes specifically express the neuronatin-α isoform.

**Fig. 2.**
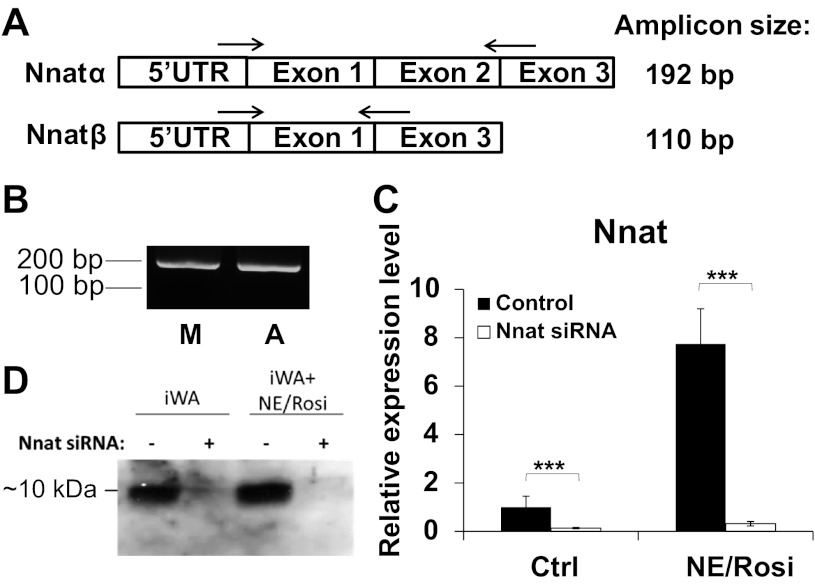
Neuronatin expression profile and silencing in murine primary adipocytes. *A*: schematic representation of the two neuronatin isoforms and the position of the primers used for their amplification. *B*: RNA isolated from muscle tissue (M) and primary white adipocyte culture (*A*) was reverse transcribed and expression of each detected by PCR. Only the longer (α) isoform (∼200 bp) was detectable. Analysis of neuronatin mRNA levels by qPCR (*C*) and protein levels by Western blot (*D*) in brown adipocyte precursors (BA) and white adipocyte precursors (iWA) with or without noreipnephrine/rosiglitazone (NE/Rosi) stimulation. Where indicated, cells had been transfected with the siRNA pool targeting neuronatin. Values in qPCR analysis represent means ± SE of 5 independent experiments. Expression levels in untreated adipocyte cultures were set in each experiment to 1.0 and other values expressed relative to this.

There are two splice variants of mature neuronatin mRNA, transcribed from either two or three exons. We used a single pair of primers designed to amplify both neuronatin isoforms and demonstrate two amplicons, corresponding to the α- and β-isoforms, after product electrophoresis (Fig. 2*A*). We were able to amplify only a single PCR product, consistent in length with the α-isoform of neuronatin, in inguinal white adipocytes, and indeed in murine muscle tissue (Fig. 2*B*).

To study the role of neuronatin in determining adipocyte phenotype, iWA and BA from 129/sv mice were cultured in the presence or absence of the sympathetic neurotransmitter norepinephrine (NE) and the PPARγ agonist rosiglitazone (Rosi). Cultures were examined by phase-contrast microscopy after 6 days of differentiation (not shown) and showed >80% of the untreated cells and ∼100% of the NE/Rosi-treated cells had a mature morphology.

Using a 20 nM pool of siRNA to transfect the untreated and the NE/Rosi-treated preadipocyte cultures, we first established that we could successfully silence neuronatin in primary cells. The pool consisted of four different siRNA sequences directed against neuronatin mRNA (Table 1). On *day 6* after seeding, we harvested the cultures and determined the mRNA and protein levels of neuronatin by qPCR (Fig. 2*C*) and Western blot (Fig. 2*D*), respectively. siRNA addition resulted in >90% loss of neuronatin mRNA expression in iWA (Fig. 2*C*) and BA (Table 2). Western blot confirmed the presence of only one neuronatin isoform (Nnat-α) and confirmed successful silencing at the protein level.

**Table 2.** Effect of Nnat knockdown on BA marker levels and glucose uptake

	BA	BA + NE/R	BA + Nnat siRNA	BA + NE/*R* + Nnat siRNA
*Nnat*	1.00 ± 0.21	0.76 ± 0.25	0.09 ± 0.03	0.03 ± 0.01
*UCP1*	1.00 ± 0.33	595 ± 245	3.17 ± 0.79	1770 ± 1249
*aP2*	1.00 ± 0.30	3.39 ± 077	1.30 ± 0.31	5.24 ± 0.93
*PPARγ*	1.00 ± 0.42	1.18 ± 0.24	2.19 ± 0.43	2.65 ± 0.47
*PGC1α*	1.00 ± 0.38	2.37 ± 0.68	1.53 ± 0.37	4.27 ± 0.45
*Cox8b*	1.00 ± 0.33	1.53 ± 0.23	1.40 ± 0.51	2.65 ± 0.52
*Cox4*	1.00 ± 0.27	1.92 ± 0.46	1.77 ± 0.50	2.51 ± 0.42
	BA	BA + 10 nM Ins	BA + Nnat siRNA	BA + 10 nM Ins + Nnat siRNA
*Glucose uptake (nmol/mg/min)*	0.15 ± 0.03	0.40 ± 0.09	0.07 ± 0.04	0.14 ± 0.05

Values represent means ± SE of at least 4 independent experiments. See text for definitions.

#### Neuronatin knockdown increases expression of markers of adipogenesis in primary adipocytes.

**Fig. 3.**
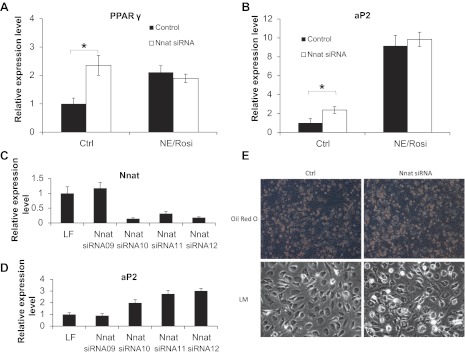
Neuronatin silencing potentiates adipogenesis in inguinal white adipocytes. Expression levels of adipogenic marker genes PPARγ (*A*) and aP2 (*B*) in control and NE/Rosi-treated inguinal white adipocytes with or without neuronatin knockdown, were measured. Expression levels are given relative to those of the BA cultures. Statistical analysis of the siRNA effects were performed and indicated as described in methods. Neuronatin (*C*) and aP2 (*D*) levels were measured in inguinal white adipocytes in the presence of individual siRNAs from the siRNA pool targeting neuronatin. Values represent means ± SE of qPCR triplicates from a single experiment normalized to 18S expression relative to levels in Lipofectamine-treated adipocyte cultures. *E*: representative phase-contrast pictures of cells with and without neuronatin knockdown and with and without Oil red O staining.

Expression of one of the major mediators of adipocyte differentiation, PPARγ, was high (C_T_ ∼23) in inguinal white adipocytes (Fig. 3*A*) and in BA (Table 2). PPARγ mRNA was somewhat increased in iWA by chronic treatment with NE/Rosi, but not significantly changed in BA under the same treatment conditions. However, PPARγ mRNA was increased upon neuronatin knockdown in iWA, reaching the level present in iWA chronically treated with NE/Rosi (Fig. 3*A*). Similar increases were seen in BA with and without treatment (Table 2). Thus, neuronatin appears to have a negative impact on PPARγ expression under a variety of conditions. PRDM16, a transcription factor thought to modulate brown adipocyte formation (29), was expressed at similar levels in all cell types and conditions, and its expression was not affected by neuronatin knockdown (data not shown).

Chronic NE/Rosi treatment, as expected (25, 26), resulted in increased expression of the adipogenic marker aP2 in both iWA (by ∼800%) and BA (by ∼300%) (Fig. 3*B* and Table 2). Baseline levels of aP2 mRNA expression were very high (C_T_ values ∼15), supporting the observation that the cells were robustly differentiated in culture. Neuronatin silencing caused a significant further increase in the expression of aP2 only in control iWA cells, although there was also a tendency for aP2 to increase under the other treatment conditions. Of the four independent siRNA sequences that constituted the siRNA pool targeting neuronatin, three were able to robustly knock down neuronatin RNA (Fig. 3*C*), and, critically, only those three led to an increased expression of the adipogenesis marker aP2 (Fig. 3*D*). This provides evidence that specific effective silencing was required to impact on phenotype and implies that off-target effects of the siRNA are less likely. To examine whether neuronatin knockdown affected the differentiation status of the cells, we studied the cell cultures by phase-contrast microscopy with and without Oil red O staining. neuronatin silencing in iWA resulted in a somewhat more mature appearance (Fig. 3*E*). However, lipid content measured upon Oil red O elution with isopropanol was not different between control and neuronatin knockdown cells (data not shown), which might suggest that the number of mature lipid-containing cells was slightly increased but the overall content of lipid was not.

#### Neuronatin silencing induces mitochondrial biogenesis in primary adipocytes.

**Fig. 4.**
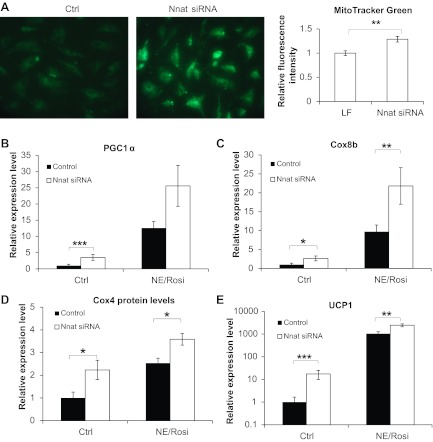
Neuronatin silencing potentiates mitochondrial biogenesis and a BAT-like phenotype in inguinal white adipocytes. Expression levels of thermogenic marker genes in control and NE/Rosi-stimulated cultures of inguinal white adipocytes were measured with and without neuronatin silencing. *A*: inguinal white adipocytes were analyzed for mitochondrial abundance by MitoTracker Green staining. Cells were examined with a fluorescence microscope, and representative images are shown. Fluorescence intensity was measured as described in methods. PGC-1α (*B*), Cox8b (*C*), and UCP1 (*E*, note logarithmic *y*-axes) mRNA levels are shown in control and NE/Rosi-treated inguinal white adipocytes with and without neuronatin siRNA pool. Expression levels in untreated adipocyte cultures were set in each experiment to 1.0, and levels in the other cultures were expressed relative to this value in each experiment. Values represent means ± SE of 5 independent experiments. *D*: protein levels of Cox4 in control and NE/Rosi-treated inguinal white adipocytes with and without neuronatin siRNA were analyzed by Western blotting, and the graph represents means ± SE of 4 independent experiments. Statistical analysis was performed as described in methods.

To assess the effect of neuronatin loss on mitochondrial biogenesis, we used MitoTracker green, a membrane potential-independent mitochondrial-specific fluorescent dye, to stain iWA cells with and without neuronatin silencing. Neuronatin loss caused visibly stronger staining in iWA (Fig. 4*A*), indicating an enhancement of mitochondrial biogenesis in these cells. Quantification of the fluorescence intensity confirmed that the fluorescence signal was stronger in iWA cells lacking neuronatin.

Consistent with this finding, loss of neuronatin impacted on PGC-1α expression in iWA. PGC-1α is a key transcriptional coregulator of oxidative metabolism and thermogenesis. Its levels were low in iWA, but its expression was increased significantly after NE/Rosi treatment (C_T_ ∼25; Fig. 4*B*). Loss of neuronatin impacted on PGC-1α expression only in untreated iWA, where it was increased significantly, suggestive of increased “browning” of iWA. Neuronatin silencing did not significantly increase the already high levels of PGC-1α in NE/Rosi-treated iWA (Fig. 4*B*) or in BA (Table 2). Induction of PGC-1α was associated with a consistent increase in mitochondrial content and drove the expression of many genes involved in mitochondrial oxidative phosphorylation (27), as indicated by measurement of Cox8b mRNA expression and Cox4 protein expression. Cox8b was expressed at a very low level in control iWA, but NE/Rosi treatment led to a very substantial increase in expression (Fig. 4*C*). However, its expression level was high in control BA and was therefore not significantly increased by NE/Rosi treatment (Table 2). Cox8b levels were increased by neuronatin silencing in both control and treated iWA, consistent with the impact of loss of neuronatin on PGC-1α levels. We also examined the effect of neuronatin silencing on protein level of Cox4 (Fig. 4*D* and Table 2) and found this to be consistent with the qPCR data, being markedly increased in iWA.

The brown/“brite”-specific mitochondrial protein UCP1 was expressed at a low level in control iWA, but upon chronic NE/Rosi treatment, UCP1 was strongly (∼700-fold) induced (Fig. 4*E*; note the logarithmic scale). UCP1 was expressed at an approximately sixfold higher level in BA and was also massively increased by NE/Rosi treatment (Table 2). Loss of neuronatin expression resulted in a significant upregulation of UCP1 levels in iWA with or without NE/Rosi treatment (Fig. 4*E*). The magnitude of the effect was largest when the baseline expression of UCP1 was lowest (Fig. 4*E* and Table 2; note the logarithmic scale).

**Fig. 5.**
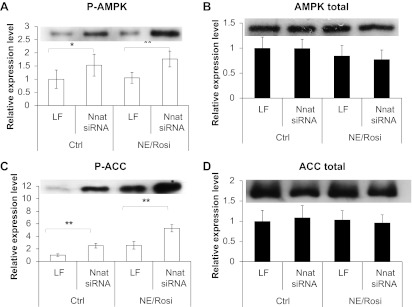
Effect of neuronatin knockdown on activation of AMPK and inactivation of ACC. Western blot analysis is shown for p-AMPK (*A*), total AMPK (*B*), p-ACC (*C*), and total ACC (*D*) in control and NE/Rosi-treated inguinal white adipocytes with and without neuronatin silencing. Graphs show means ± SE of 4–6 independent experiments. Representative blots are shown for each protein. Statistical analysis was conducted as described in methods.

Notably, neuronatin silencing led to significantly increased phosphorylation of AMP-activated protein kinase (AMPK) at Thr^172^ (Fig. 5*A*), without any effect on total levels of AMPK (Fig. 5*B*). Acetyl-CoA carboxylase-1 (ACC1) phosphorylation at Ser^79^ was also increased by neuronatin silencing, without effect on total ACC1 (Fig. 5, *B* and *C*). AMPK-mediated phosphorylation of ACC1 at Ser^79^ would be expected to result in reduced formation of malonyl-CoA and, hence, promote greater carnitine palmitoyltransferase-1 (CPT I)-mediated mitochondrial fatty acid uptake.

#### Neuronatin silencing impairs glucose uptake and glycogen synthesis.

In iWA, insulin induced an approximately twofold increase in glucose uptake over basal (Fig. 5*A*). A similar increase was seen in BA (Table 2). Neuronatin knockdown in iWA led to significantly decreased glucose uptake, primarily in the basal state but also during insulin stimulation (Fig. 5*A*). In BA, the effect of neuronatin knockdown was significant only in the insulin-stimulated state. However, the fold induction by insulin was not significantly different between control and insulin-stimulated conditions either in iWA or in BA. Insulin markedly increased glycogen synthesis in iWA, an effect that was markedly decreased by neuronatin silencing (Fig. 5*B*). As above, the fold induction by insulin was not significantly different between control and insulin-stimulated conditions. As impaired glucose uptake may have been the result of inhibition of glucose oxidation at the level of the pyruvate dehydrogenase complex (PDC), we examined whether the PDC activator dichloroacetate (DCA) (36) could relieve the inhibition of glucose uptake following loss of neuronatin expression. However, no impact of DCA on glucose uptake was observed (*n* = 2; data not shown).

#### Effect of neuronatin silencing on mediators of glucose disposal.

**Fig. 6.**
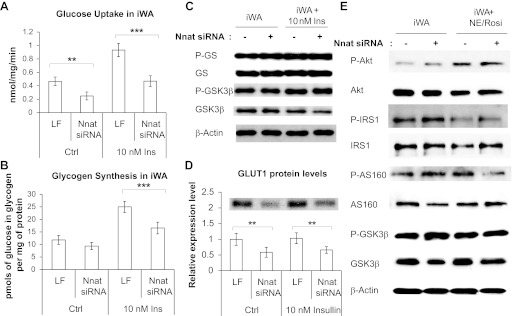
Effect of neuronatin silencing on glucose uptake and glycogen synthesis in primary adipocytes. *A*: glucose uptake in inguinal white primary adipocytes with or without insulin stimulation and with or without neuronatin siRNA. Values represent means ± SE of 5 independent experiments, each conducted in triplicate. *B*: effect of neuronatin silencing on glycogen synthesis in control and insulin-stimulated inguinal white adipocyte cultures. Values represent means ± SE of 4 independent experiments, each conducted in triplicate (pmoles of glucose incorporated in glycogen per mg of protein). *C*: Western blots shown for P-GS, GS, P-GSK3β, and GSK3β with and without insulin stimulation of iWA are representative of 4 independent experiments. *D*: Western blot analysis is shown for GLUT1 with and without insulin stimulation; graphs represent means ± SE of 4 independent experiments. A representative blot is shown above the graph. Statistical analysis was conducted as described in methods. *E*: representative Western blots (minimum 3 independent experiments) are shown for principal insulin signaling intermediates upon neuronatin knockdown with or without NE/Rosi stimulation.

We next examined whether altered signaling events might be responsible the impairment in glucose disposal caused by loss of neuronatin. Western blot analysis of protein extracts from iWA (±insulin stimulation) showed no impact of loss of neuronatin on phosphorylation of downstream susbstrates directly involved in insulin-stimulated glycogen synthesis, namely GS and GSK3β, was unaffected (Fig. 6*C*) under basal or insulin-stimulated conditions. We also obtained the same results with brown adipocytes (data not shown).

As the principal effect of neuronatin silencing was on basal glucose uptake and phosphorylation of insulin signaling intermediates was unaffected, we examined the effect of silencing on GLUT1, the principal glucose transporter for basal glucose uptake (Fig. 6*D*). GLUT1 expression was significantly decreased upon neuronatin loss under both control and insulin-stimulated conditions.

To verify that the insulin signaling pathway was not affected either in untreated or chronically NE/Rosi-stimulated adipocytes, we performed Western blot analysis of protein extracts from iWA (±chronic NE/Rosi stimulation) and showed no impact of loss of neuronatin on phosphorylation of some of the major components of the insulin signaling (phosphoinositol 3-kinase) pathway (Fig. 6*E*).

## DISCUSSION

Neuronatin is emerging as a potentially interesting obesity gene, as mutations at the gene are associated with severe forms of obesity. Recently, the protein has also been linked to a novel pathway regulating glycogen metabolism, providing further context to suggest that neuronatin may contribute to the regulation of human metabolism. In the present study, we noted that neuronatin expression is reduced in adipose tissue in obese humans and that loss of neuronatin in primary adipocytes substantially impairs basal glucose uptake and glycogen synthesis. We also provide evidence that neuronatin may contribute to the regulation of adipocyte phenotype, since it acts as a negative regulator of the “brite” gene expression program in adipocytes from murine iWA.

### 

#### Neuronatin-α regulates glucose disposal in primary adipocytes.

The expression pattern of the neuronatin gene in primary adipocytes has not been previously studied. However, current evidence suggests that each may have distinct functions. Here, we have demonstrated that only the larger, α-isoform is expressed in primary mouse adipocytes. Although neuronatin mRNA levels were somewhat lower in iWA than in BA, the expression of neuronatin protein appeared similar in the two adipocyte preparations. These preparations inevitably contained some cells derived from other lineages and our initial measurement in humans was made on whole adipose tissue; thus, it is possible that neuronatin protein also plays a role in other components of the stromal-vascular fraction of adipose tissue. However, nonadipocyte contamination was low, as can be seen in Fig. 3, whereas the magnitude of change in insulin-stimulated glucose disposal as a result of neuronatin silencing clearly implicates an insulin-sensitive cell type. Although it is not yet clear which factors regulate the expression pattern of neuronatin in adipocytes, bioinformatic analysis of the neuronatin promoter has identified conserved binding sites for transcription factors concerned with the control of adipogenesis, lipogenesis, inflammation, insulin signaling, and type 2 diabetes susceptibility (17). Thus, it also appears logical that many pathways known to be modulated as a consequence of obesity may also impact on the expression of neuronatin.

The regulation of glycogen metabolism is complex, and recent evidence points to a variety of proteins being associated with the glycogen macromolecule (7), which provide feedback cues to the glucose uptake and glycogen synthesis machinery. Notably, a failure of these proteins to be tightly regulated can result in a variety of glycogen storage diseases (7, 30, 31). For example, malin (NHLRC1/EPM2B gene) is an E3 ubiquitin ligase that, together with the phosphatase laforin (EPM2A gene), helps target GS and glycogen debranching enzyme for proteasomal degradation (30). siRNA-mediated knockdown of malin resulted in increased glucose uptake and more glucose transporters being located to the plasma membrane (31). Sharma et al. (30) recently reported that neuronatin was a substrate of malin and that malin was responsible for regulating the degradation of neuronatin in PC12 cells. Thus, the trio of malin, laforin, and neuronatin appears to be a glucose-sensing mechanism for controlling cellular glycogen metabolism and a new pathway linked to long-term modification of the glycogen macromolecule.

In this study, we have demonstrated for the first time in metabolically important cells, that silencing of neuronatin reduces both basal and insulin-stimulated glucose uptake and glycogen synthesis without any obvious impact on insulin signaling. We have shown a significant decrease of GLUT1 protein upon neuronatin loss, which likely mediates this effect on glucose uptake. Reduced glycogen synthesis may thus be a mass action effect, as a consequence of the reduced concentration of glucose within the cell, since no impact of neuronatin silencing on phosphorylation of GSK3β or GS was detected. It has previously been shown in primary brown adipocytes that NE induces a potent increase in GLUT1 mRNA and a decrease of GLUT4 mRNA (3). Dallner et al. also showed that adrenergic stimulation induces glucose uptake in brown adipocytes via a mechanism independent of GLUT1 and GLUT4 translocation but instead involving de novo synthesis of GLUT1 protein and increase of GLUT1 protein at the plasma membrane. It is possible that neuronatin is involved in this mechanism. However, as its effects on glucose disposal are consistent with those of adrenergic stimulation with respect to glucose disposal while being opposite to those on adipocyte “browning”, it would appear that it regulates each of these processes through independent mechanisms.

The impaired glucose uptake and glycogen synthesis was accompanied by an increase in the phosphorylation of AMPK and the predictable inactivation of ACC by phosphorylation. This may represent an attempt to promote greater mitochondrial fatty acid uptake to compensate for a decrease in glucose uptake and thus de novo lipogenesis. This implies that lower neuronatin expression, such as we observed in obese subjects, could contribute to obesity-induced alterations in glycogen and lipid metabolism. It is also important to reflect on the fact that the alterations in substrate utilization noted in our cultured systems are not predictive of the net effect in vivo. This is because loss of the substrate storage facilitation by neuronatin is likely to be offset by higher glucose oxidation if the “brite” adipocytes are also subject to hormone induced uncoupling.

#### Neuronatin is a negative regulator of the brown or “brite” adipocyte gene expression program.

Our microarray analysis showed that neuronatin expression in human subcutaneous adipose tissue was lower in obese individuals, while UCP1 levels followed the opposite pattern, albeit that expression of the two mRNAs was not correlated. In addition, an association between single nucleotide polymorphisms in the human neuronatin gene locus and severe obesity has been demonstrated in both children and adults (39). Interestingly, neuronatin expression in WAT and aortic endothelial cells is increased in obese and diabetic rodents (22, 32). In contrast, neuronatin expression in pancreatic β-cell lines is reduced in diabetic rodents (1). Thus, it seems that obesity may be associated with tissue-specific changes in neuronatin expression, which may relate to the neuronatin isoform that is preferentially expressed in each, although more research will be necessary before conclusions can be drawn concerning naturally occurring metabolic disease. However, one could speculate, based on our findings, that neuronatin downregulation may be an adaptive mechanism aimed at limiting triglyceride accumulation and thus increasing adiposity through increased mitochondrial fatty acid oxidation and reduced de novo lipogenesis.

In this study, neuronatin mRNA expression was highly induced by chronic in vitro treatment with a PPARγ agonist in iWA, to levels even greater than those found in untreated BA (Fig. 2*C*), whereas neuronatin mRNA was not induced in BA by the same treatment. Although different pathways may be modulated by NE/Rosi treatment in these two types of cells (40), our observations suggest that neuronatin may be involved in “brite” cell differentiation. Furthermore, loss of neuronatin expression resulted in the induction of Cox genes and UCP1 in iWA, implying that it may be a negative regulator of the adipocyte “browning” process. This phenotype of increased mitochondrial markers and ACC phosphorylation, accompanied by reduced glucose uptake, is suggestive of a shift toward increased and preferential oxidation of fatty acids in adipocytes lacking neuronatin, although this hypothesis requires confirmation through more definitive study.

Neuronatin has also been proposed to mediate its effects through modulation of cellular calcium flux, with consequent effects on activation of downstream signaling pathways, including extracellular signal-regulated kinase (18, 24). It has been reported that overexpression of the α-isoform resulted in phosphorylation and therefore likely increased transcriptional activation by cAMP-response element-binding protein (CREB) in adipocytes (33). However, CREB inhibition in brown adipocytes has been shown to result in reduced UCP1 expression (21, 23). Given that we demonstrate that loss of neuronatin potentiates, rather than compromises, induction of UCP1 and mitochondrial genes, this might suggest that neuronatin regulates transcriptional programs downstream of calcium flux.

Currently, thiazolidinediones (TZDs) represent the only pharmacological tool that robustly activates a full “brite” gene expression program, but in humans these drugs also promote weight gain and demonstrate other undesirable side effects (9). Nevertheless, use of low-dose rosiglitazone has been considered to be potentially protective against cardiovascular disease; hence, its biological effects are still highly relevant (20). The discovery that loss of neuronatin can promote UCP1 induction is a potentially important discovery, and more detailed analysis of neuronatin biochemistry is required before it can be proposed that manipulation of neuronatin represents a point for therapeutic intervention in human obesity.

## GRANTS

This study was supported by a grant from the BBSRC, UK. M. E. Cleasby is supported by a Wellcome Trust University Award.

## DISCLOSURES

No conflicts of interest, financial or otherwise, are declared by the author(s).

## AUTHOR CONTRIBUTIONS

Author contributions: V.G., M.E.C., and J.A.T. conception and design of research; V.G. performed experiments; V.G. analyzed data; V.G., M.E.C., and J.A.T. interpreted results of experiments; V.G. prepared figures; V.G. and J.A.T. drafted manuscript; V.G., M.E.C., and J.A.T. approved final version of manuscript; M.E.C. and J.A.T. edited and revised manuscript.

## References

[1] Chu K Tsai MJ Neuronatin, a downstream target of BETA2/NeuroD1 in the pancreas, is involved in glucose-mediated insulin secretion. Diabetes 54: 1064–1073, 2005 15793245 10.2337/diabetes.54.4.1064 PMC1197706

[2] Cleasby ME Davey JR Reinten TA Graham MW James DE Kraegen EW Cooney GJ Acute bidirectional manipulation of muscle glucose uptake by in vivo electrotransfer of constructs targeting glucose transporter genes. Diabetes 54: 2702–2711, 2005 16123360 10.2337/diabetes.54.9.2702

[3] Dallner OS Chernogubova E Brolinson KA Bengtsson T Beta3-adrenergic receptors stimulate glucose uptake in brown adipocytes by two mechanisms independently of glucose transporter 4 translocation. Endocrinology 147: 5730–5739, 2006 16959848 10.1210/en.2006-0242

[4] Dou D Joseph R Structure and organization of the human neuronatin gene. Genomics 33: 292–297, 1996 8660979 10.1006/geno.1996.0195

[5] Gburcik V Cawthorn WP Nedergaard J Timmons JA Cannon B An essential role for Tbx15 in the differentiation of brown and “brite” but not white adipocytes. Am J Physiol Endocrinol Metab 303: E1053–E1060, 2012 22912368 10.1152/ajpendo.00104.2012

[6] Goodpaster BH Thaete FL Simoneau JA Kelley DE Subcutaneous abdominal fat and thigh muscle composition predict insulin sensitivity independently of visceral fat. Diabetes 46: 1579–1585, 1997 9313753 10.2337/diacare.46.10.1579

[7] Graham TE Yuan Z Hill AK Wilson RJ The regulation of muscle glycogen: the granule and its proteins. Acta Physiol (Oxford) 199: 489–498, 2010 10.1111/j.1748-1716.2010.02131.x 20353490

[8] Guerra C Koza RA Yamashita H Walsh K Kozak LP Emergence of brown adipocytes in white fat in mice is under genetic control. Effects on body weight and adiposity. J Clin Invest 102: 412–420, 1998 9664083 10.1172/JCI3155 PMC508900

[9] Hauner H The mode of action of thiazolidinediones. Diabetes Metab Res Rev 18, Suppl 2: S10–S15, 2002 11921433 10.1002/dmrr.249

[10] Hegarty BD Furler SM Ye J Cooney GJ Kraegen EW The role of intramuscular lipid in insulin resistance. Acta Physiol Scand 178: 373–383, 2003 12864742 10.1046/j.1365-201X.2003.01162.x

[11] Helge JW Stallknecht B Drachmann T Hellgren LI Jiménez-Jiménez R Andersen JL Richelsen B Bruun JM Improved glucose tolerance after intensive life style intervention occurs without changes in muscle ceramide or triacylglycerol in morbidly obese subjects. Acta Physiol (Oxf) 201: 357–364, 2011 20726847 10.1111/j.1748-1716.2010.02180.x

[12] Joe MK Lee HJ Suh YH Han KL Lim JH Song J Seong JK Jung MH Crucial roles of neuronatin in insulin secretion and high glucose-induced apoptosis in pancreatic beta-cells. Cell Signalling 20: 907–915, 2008 18289831 10.1016/j.cellsig.2008.01.005

[13] Joseph R Tsang W Dou D Nelson K Edvardsen K Neuronatin mRNA in PC12 cells: downregulation by nerve growth factor. Brain Res 738: 32–38, 1996 8949924 10.1016/0006-8993(96)00768-8

[14] Keller P Gburcik V Petrovic N Gallagher IJ Nedergaard J Cannon B Timmons JA Gene-chip studies of adipogenesis-regulated microRNAs in mouse primary adipocytes and human obesity. BMC Endocr Dis 11: 7, 2011 10.1186/1472-6823-11-7 PMC3070678 21426570

[15] Kikyo N Williamson CM John RM Barton SC Beechey CV Ball ST Cattanach BM Surani MA Peters J Genetic and functional analysis of neuronatin in mice with maternal or paternal duplication of distal Chr 2. Dev Biol 190: 66–77, 1997 9331332 10.1006/dbio.1997.8681

[16] Kozak LP Anunciado-Koza R UCP1: its involvement and utility in obesity. Int J Obes (Lond) 32, Suppl 7: S32–S38, 2008 19136989 10.1038/ijo.2008.236 PMC2746324

[17] Li X Thomason PA Withers DJ Scott J Bio-informatics analysis of a gene co-expression module in adipose tissue containing the diet-responsive gene Nnat. BMC Syst Biol 4: 175, 2010 21187013 10.1186/1752-0509-4-175 PMC3022651

[18] Lin HH Bell E Uwanogho D Perfect LW Noristani H Bates TJ Snetkov V Price J Sun YM Neuronatin promotes neural lineage in ESCs via Ca(2+) signaling. Stem Cells 28: 1950–1960, 2010 20872847 10.1002/stem.530 PMC3003906

[19] Livak KJ Schmittgen TD Analysis of relative gene expression data using real-time quantitative PCR and the 2(-Delta Delta C_T_) method. Methods (San Diego, Calif) 25: 402–408, 2001 10.1006/meth.2001.1262 11846609

[20] Lonn EM Gerstein HC Sheridan P Smith S Diaz R Mohan V Bosch J Yusuf S Dagenais GR Effect of ramipril and of rosiglitazone on carotid intima-media thickness in people with impaired glucose tolerance or impaired fasting glucose: STARR (STudy of Atherosclerosis with Ramipril and Rosiglitazone). J Am Coll Cardiol 53: 2028–2035, 2009 19477351 10.1016/j.jacc.2008.12.072

[21] Muraoka M Fukushima A Viengchareun S Lombès M Kishi F Miyauchi A Kanematsu M Doi J Kajimura J Nakai R Uebi T Okamoto M Takemori H Involvement of SIK2/TORC2 signaling cascade in the regulation of insulin-induced PGC-1α and UCP-1 gene expression in brown adipocytes. Am J Physiol Endocrinol Metab 296: E1430–E1439, 2009 19351809 10.1152/ajpendo.00024.2009

[22] Mzhavia N Yu S Ikeda S Chu TT Goldberg I Dansky HM Neuronatin: a new inflammation gene expressed on the aortic endothelium of diabetic mice. Diabetes 57: 2774–2783, 2008 18591389 10.2337/db07-1746 PMC2551689

[23] Nedergaard J Golozoubova V Matthias A Asadi A Jacobsson A Cannon B UCP1: the only protein able to mediate adaptive non-shivering thermogenesis and metabolic inefficiency. Biochim Biophys Acta 1504: 82–106, 2001 11239487 10.1016/s0005-2728(00)00247-4

[24] Oyang EL Davidson BC Lee W Poon MM Functional characterization of the dendritically localized mRNA neuronatin in hippocampal neurons. PloS one 6: e24879, 2011 21935485 10.1371/journal.pone.0024879 PMC3173491

[25] Petrovic N Shabalina IG Timmons JA Cannon B Nedergaard J Thermogenically competent nonadrenergic recruitment in brown preadipocytes by a PPARγ agonist. Am J Physiol Endocrinol Metab 295: E287–E296, 2008 18492776 10.1152/ajpendo.00035.2008

[26] Petrovic N Walden TB Shabalina IG Timmons JA Cannon B Nedergaard J Chronic peroxisome proliferator-activated receptor gamma (PPARgamma) activation of epididymally derived white adipocyte cultures reveals a population of thermogenically competent, UCP1-containing adipocytes molecularly distinct from classic brown adipocyt. J Biol Chem 285: 7153–7164, 2010 20028987 10.1074/jbc.M109.053942 PMC2844165

[27] Puigserver P Spiegelman BM Peroxisome proliferator-activated receptor-gamma coactivator 1 alpha (PGC-1 alpha): transcriptional coactivator and metabolic regulator. Endocr Rev 24: 78–90, 2003 12588810 10.1210/er.2002-0012

[28] Saltiel AR New perspectives into the molecular pathogenesis and treatment of type 2 diabetes. Cell 104: 517–529, 2001 11239409 10.1016/s0092-8674(01)00239-2

[29] Seale P Kajimura S Yang W Chin S Rohas LM Uldry M Tavernier G Langin D Spiegelman BM Transcriptional control of brown fat determination by PRDM16. Cell Metab 6: 38–54, 2007 17618855 10.1016/j.cmet.2007.06.001 PMC2564846

[30] Sharma J Rao SNR Shankar SK Satishchandra P Jana NR Lafora disease ubiquitin ligase malin promotes proteasomal degradation of neuronatin and regulates glycogen synthesis. Neurobiol Dis 44: 133–41, 2011 21742036 10.1016/j.nbd.2011.06.013

[31] Singh PK Singh S Ganesh S The laforin-malin complex negatively regulates glycogen synthesis by modulating cellular glucose uptake via glucose transporters. Mol Cell Biol 32: 652–663, 2012 22124153 10.1128/MCB.06353-11 PMC3266598

[32] Suh YH Kim WH Moon C Hong YH Eun SY Lim JH Choi JS Song J Jung MH Ectopic expression of Neuronatin potentiates adipogenesis through enhanced phosphorylation of cAMP-response element-binding protein in 3T3-L1 cells. Biochem Biophys Res Commun 337: 481–489, 2005 16223607 10.1016/j.bbrc.2005.09.078

[33] Suh YH Kim WH Moon C Hong YH Eun SY Lim JH Choi JS Song J Jung MH Ectopic expression of Neuronatin potentiates adipogenesis through enhanced phosphorylation of cAMP-response element-binding protein in 3T3–L1 cells. Biochem Biophys Res Commun 337: 481–489, 2005 16223607 10.1016/j.bbrc.2005.09.078

[34] Summers SA Sphingolipids and insulin resistance: the five Ws. Curr Opin Lipidol 21: 128–135, 2010 20216312 10.1097/MOL.0b013e3283373b66

[35] Sun K Kusminski CM Scherer PE Adipose tissue remodeling and obesity. J Clin Invest 121: 2094–2101, 2011 21633177 10.1172/JCI45887 PMC3104761

[36] Timmons JA Constantin-Teodosiu D Poucher SM Greenhaff PL Acetyl group availability influences phosphocreatine degradation even during intense muscle contraction. J Physiol 561: 851–859, 2004 15498812 10.1113/jphysiol.2004.069419 PMC1665386

[37] Timmons JA Pedersen BK The importance of brown adipose tissue. N Engl J Med 361: 415–421, 2009 19625723 10.1056/NEJMc091009

[38] Tung YC Ma M Piper S Coll A O'Rahilly S Yeo GS Novel leptin-regulated genes revealed by transcriptional profiling of the hypothalamic paraventricular nucleus. J Neurosci 28: 12419–12426, 2008 19020034 10.1523/JNEUROSCI.3412-08.2008 PMC2650686

[39] Vrang N Meyre D Froguel P Jelsing J Tang-Christensen M Vatin V Mikkelsen JD Thirstrup K Larsen LK Cullberg KB Fahrenkrug J Jacobson P Sjöström L Carlsson LM Liu Y Liu X Deng HW Larsen PJ The imprinted gene neuronatin is regulated by metabolic status and associated with obesity. Obesity (Silver Spring) 18: 1289–1296, 2010 19851307 10.1038/oby.2009.361 PMC2921166

[40] Waldén TB Hansen IR Timmons JA Cannon B Nedergaard J Recruited vs nonrecruited molecular signatures of brown, “brite,” and white adipose tissues. Am J Physiol Endocrinol Metab 302: E19–E31, 2012 21828341 10.1152/ajpendo.00249.2011

